# Shake the Disease. Georges Marinesco, Paul Blocq and the Pathogenesis of Parkinsonism, 1893

**DOI:** 10.3389/fnana.2016.00074

**Published:** 2016-06-24

**Authors:** Sorin Hostiuc, Eduard Drima, Octavian Buda

**Affiliations:** ^1^National Institute of Legal Medicine Bucharest, Carol Davila University of Medicine and PharmacyBucharest, Romania; ^2^Galați Psychiatry Hospital, University of Medicine and PharmacyGalați, Romania

**Keywords:** Parkinson’s disease, shaking palsy, substantia nigra, Georges Marinesco, Paul Blocq

## Abstract

James Parkinson, in his “Essay on the Shaking Palsy” from 1817 described for the first time the disease that later on carried his name. Its anatomical substrate remained controversial for over 100 years. The first case that suggested the association between Parkinson’s disease and substantia nigra was published in 1893 Blocq and Marinesco, two scientists who worked at Salpêtrière. The article described a 38 years-old man, with tuberculosis, who was admitted to the Charcot’s neurological ward because he also showed signs of unilateral Parkinsonism. During the autopsy, the investigators found a tubercle that destroyed the right substantia nigra. As the patient had overactive reflexes on the left side and the symptomatology matched exactly the localization of the tumor, Blocq and Marinesco suggested the Parkinsonism to be more likely a complication of tuberculosis and not an incidental finding. In this article, we will discuss the contribution of these two authors to the elucidation of the pathology of Parkinson’s disease, and highlight how even a single case report may play an essential role in the development of knowledge in biomedical sciences.

## Introduction

Parkinson’s disease, nowadays one of the most frequent and debilitating neurological disorders (Pringsheim et al., 2014), was described for the first time at the beginning of the 19th century when James Parkinson published his “Essay on the Shaking Palsy” (Parkinson, 1817). In it, Parkinson defined the disorder as the “involuntary tremulous motion, with lessened muscular power, in parts not in action and even when supported; with a propensity to bend the trunk forward, and to pass from walking to a running pace; the senses and intellects being uninjured” (Parkinson, 1817). Parkinson’s essay cited a few predecessors who described the disease, including Galen, Sylvius de la Boe, Junker, or Cullen (Parkinson, 1817). All cases presented by those authors were not typical for what we know today as Parkinson’s disease; also, none of them described the clinical aspects of the disease in such detail as he did. The development of knowledge about Parkinson’s disease followed four main stages (Khalil, 1996). The first one was clinical, initiated by Parkinson’s essay and finished by the description of the rigidity as the second element of the symptomatic triad in the 1880’s. The second stage was neuro-pathological. It started with the inquiries shepherded by Charcot (1887) and his pupils, and ended with the works of Tretiakoff—who proved the association between Parkinson’s disease and substantia nigra, and of Foix and Nicolesco (1925), Hassler (1938), Greenfield and Bosanquet (1953) who showed that the changes in the pallidum are secondary. The third stage—neurophysiological and neurosurgical, began with Wilson’s cortical resection (Khalil, 1996) while the fourth was biochemical—the stage of therapeutic applications.

This article presents the case that most authors consider being the first piece of information that led to the hypothesis about the involvement of substantia nigra in the pathology of Parkinson’s disease, and to summarize its consequences for the development of knowledge in this field.

### Neuropathological Findings and Theories Before 1893

Parkinson initially suggested that the anatomical location of the lesions causing the “shaking palsy” was the spinal cord: “All that had been ventured to assume here, has been that the disease depends on a disordered state of the medulla, which is contained in the cervical vertebrae. But of what nature that morbid change is; and whether originating in the medulla itself, in its membranes, or in the containing theca, is; at present, the subject of doubt and conjecture” (Parkinson, 1817).

Rudolf Leubuscher, a famous German neurologist, and a promoter of the health reform in Germany at the middle of the 19th century reported a case in which he found an association between tremors and a fibrous tumor of the pons (Leubuscher, 1860; Ordenstein, 1868). Leopold Ordenstein, a German neurophysiologist, and the first author to adequately differentiate Parkinson’s disease from multiple sclerosis (Lehmann et al., 2007) described a softening of the substantia nigra in a patient with tremors but did not speculate upon its importance (Ordenstein, 1868). Hughlings Jackson considered that the morphological substrate of the disease was in the cerebellum; he suggested a distinctive antagonism between the cerebellar and cerebral influences, the latter causing flexion of the phalangeal joints and the former—flexion of the metacarpophalangeal joints (Gowers, 1896). Gowers (1896) believed that Jackson’s theory was unfounded. He considered that the symptoms are more likely caused by an un-antagonized cerebral influence, as “the causes in which tremor results from organic disease of the nervous system, we find situated, as a rule, within the cerebral hemispheres, in the optic thalamus, posterior part of the internal capsule, foot of the corona radiata (Nothnagel), parietal lobe of the cortex (Chvostek), and the island of Reil (Leyden)” (Gowers, 1896).

A big step forward in the understanding of this disease came from the works of Charcot (1879) at the Salpêtrière Hospital in Paris. The works of Charcot’s and his pupils aided in the differentiation of Parkinson’s disease from other neurological disorders with associated tremor such as multiple sclerosis (Charcot, 1879; Lehmann et al., 2007). Charcot described a series of symptoms that were, as he commented, overlooked by Parkinson, as the rigidity of the neck, trunk and extremities (Charcot, 1879). They also showed that tremor, bradykinesia, balance impairment, and rigidity are the four cardinal symptoms of the disease (Gowers, 1896). Charcot was the one who coined the term Parkinson’s disease, considering the term paralysis agitans to be improper (Parent and Parent, 2010). However, he felt that Parkinson’s disease is a neurosis, without a proper structural cause (Pearce, 1989), an opinion shared by Babinski or Brissaud. In this neuropathology school at Salpêtrière worked, amongst others, Georges Marinesco, Paul Blocq, Edouard Brissaud or Konstantin Tretiakoff, all involved in the discovery of the pathological substrate of the disease.

Blocq (1860–1896) was a French neuropathology researcher from the school of Charcot at Salpêtrière. He contributed, amongst others, to the characterization of the astasia-abasia syndrome (now known as Blocq’s disease; Ordenstein, 1868), or neurasthenia and related diseases (Blocq, 1891a,b,c). His major works were done conjointly with Georges Marinesco and included a study on the pathogeny of essential epilepsy (Blocq and Marinesco, 1892) where they described lesions now known as senile plaques (Buda et al., 2009) and a famous Atlas of the Histopathology of the Nervous System (Blocq et al., 1892). He died young, at the age of 36.

Georges Marinesco (1863–1938, Figure 1) was one of the most influential Romanian and European neuroscientists at the beginning of the 20th century (Buda et al., 2013). At the end of the 19th century, while undertaking postgraduate courses of neurology at Salpêtrière under the supervision of Jean-Martin Charcot, he worked with some of the most prominent neurologists or neuropathologists of that time including Joseph Babinski, Pierre Marie, or Paul Blocq (Buda et al., 2009). In the last decade of the 19th century, he developed an interest for neurodegeneration, area in which, together with Blocq, he had outstanding contributions to the portrayal of Alzheimer’s disease, Friederich’s ataxia, or Parkinson’s disease.

**Figure 1:**
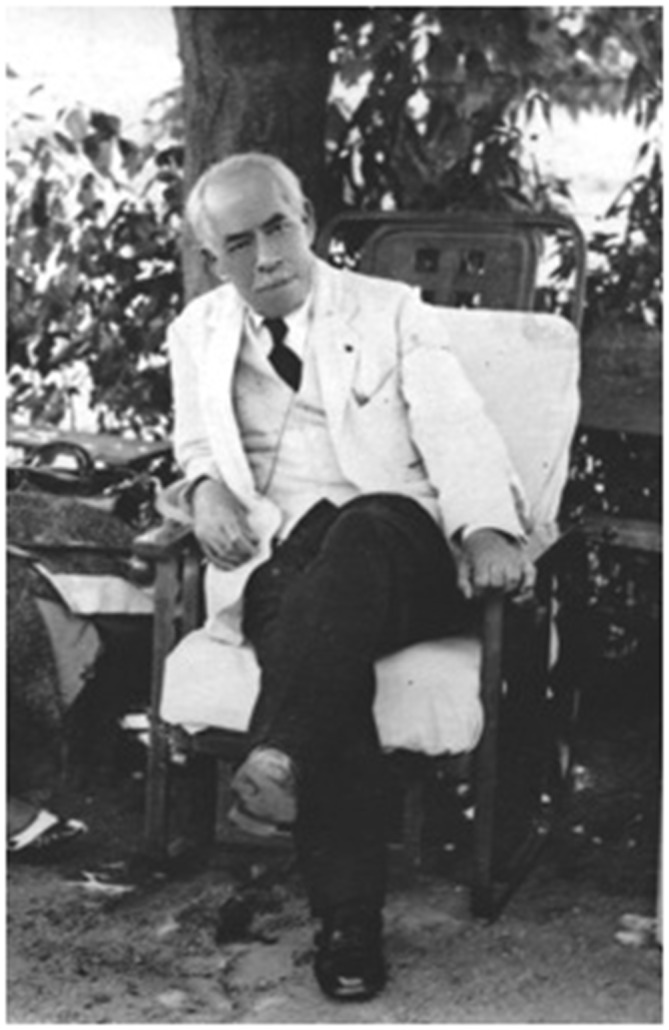
**Georges Marinesco, in the 20’s.** Personal photo collection of author 3.

**Figure 2:**
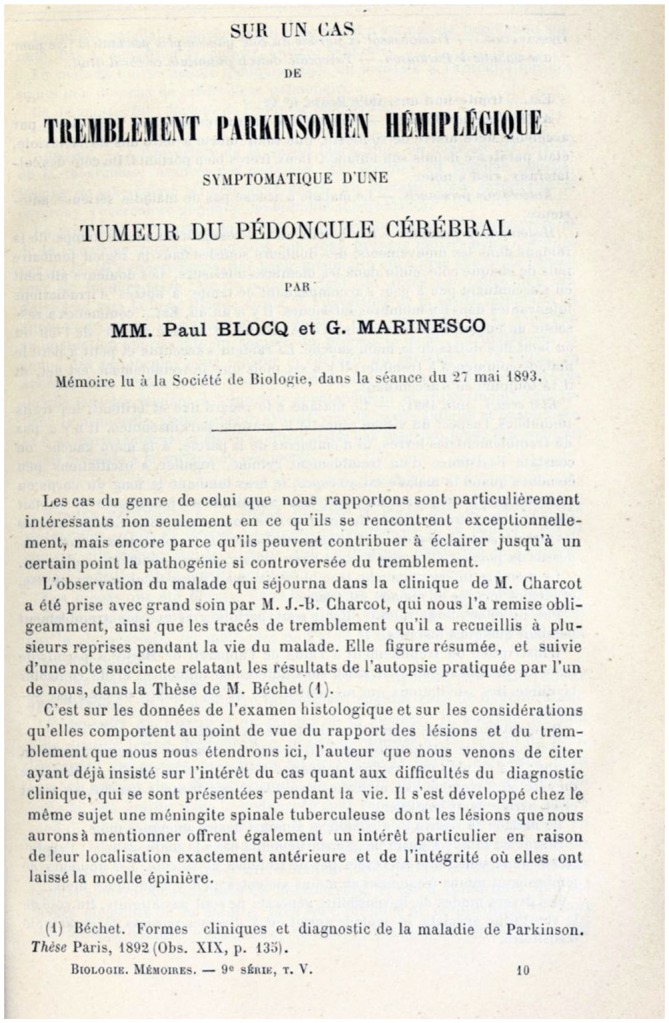
**First page of the article.** Public domain (article published in 1892).

Maybe the most influential work published by Paul Blocq and Georges Marinesco was an article published in 1893 (Figure 2). It depicted the case of a 38 years-old man, suffering from tuberculosis, who was admitted to the Charcot’s neurological ward because he also displayed signs of unilateral Parkinsonism (primarily muscle rigidity and left side tremor). Jean-Baptiste Charcot, the son of Jean Martin Charcot, treated the patient and detailed his clinical evolution (Parent and Parent, 2010). The autopsy showed the cause of death to be pulmonary complications of tuberculosis. While examining the brain, the investigators found a tubercle that destroyed the right substantia nigra: “*Its limits in the peduncle were: in front, by the foot of the peduncle, behind by the superior cerebellar peduncle, inward by the fibers of the common oculomotorius nerve, and outward, until the elements of the medial lemniscus (Untere-Schleife). Summarizing, the tumor affected the substance of Soemmering. It is to be remarked that various elements of the protuberance, besides being displaced and compressed, were not destroyed, as it was proven by the absence of degeneration of the pyramids at the base, and of the cerebellar peduncle, at the top*.” (Blocq and Marinesco, 1893).

As the patient had overactive reflexes on the left side and the symptomatology matched exactly the localization of the tumor, Blocq and Marinesco suggested the Parkinsonism to be more likely a complication of tuberculosis and not an incidental finding. To support this hypothesis they cited relevant scientific literature. For example, in an article published by Mendel in Berliner Klinische Wochenschrift from 1885, the author described the case of a four and a half-year-old child with intentional tremor on the right arm, weakness of the right ankle, paresis of the third pair of cranial nerves, the facial, and the hypoglossal. The autopsy, in that case, revealed a tubercle on the middle part of the cap of the left cerebral peduncle, of the size of an almond, just beneath the subthalamus (Blocq and Marinesco, 1893). In that case, the tremor was intentional (intention tremor), which Charcot (1879) previously associated with peduncle lesions. He also described a patient who had a tumor compressing the cerebral peduncles, with associated resting tremor and a typical, Parkinsonian pose of the upper limbs (Blocq and Marinesco, 1893). What Blocq and Marinescu did not do, was to pinpoint the substantia nigra as the potential area involved in the progress of the disease. This hypothesis was raised only a few years later by Brissaud (1895). He analyzed the case presented by Blocq and Marinesco, emphasized that the substantia nigra was destroyed, and suggested the possibility it may be involved in muscle tone and may be the anatomical substrate for Parkinson’s disease (Brissaud, 1895). That statement received little consideration in the ensuing decades, most pathology studies in the area focusing on the striatum or the basal ganglia (especially lenticular fasciculus and ansa lenticularis) (Parent and Parent, 2010). In France, Compin (1902) supported, at the beginning of the 20th century, the model about the nigral substrate of Parkinson’s disease. Most researchers viewed this theory with circumspection and was presented in scientific articles as a form of respect for Brissaud, who coined it. The most plausible explanation at the time was that Parkinson’s disease is not a single, clearly defined disease but a syndrome, with different etiologies (traumatic, rheumatic, infectious, psychiatric) (Compin, 1902). Another cause for the fall into forgetfulness of the 1893 article was that Blocq supported another theory about the pathogeny of Parkinson’s disease. He was one of the most preeminent supporters of the myopathic theory, after identifying what he considered distinguishing muscular lesions in patients with this ailment (Fleury, 1904).

The article of Blocq and Marinesco was vastly circulated in the first decade of the 20th century. Friederich H. Lewy, the father of Lewy bodies, referenced it in an influential work from 1912: “concerning the similar structure of the cells, this could be one coherent aggregation of cells reaching from substantia nigra (Blocq-Marinesco found it destroyed by a tumor in a case of shaking palsy)” (Lewy, 1912). As it is evident Lewy knew the article, it remains a mystery why he did not search eosinophilic bodies in substantia nigra until after the article of Tretiakoff (Lewy, 1923).

The confirmation of Brissaud’s theory came in 1919, with the thesis of Konstantin Tretiakoff. He worked at Salpêtrière in the same laboratory with Georges Marinesco, who most likely suggested the topic of this thesis (Lees et al., 2008). There are no proofs to show that Blocq and Marinesco’s article led him to examine the substantia nigra in Parkinson’s disease. Most likely the suggestion of the theme was not made unambiguously for Tretiakoff to explain the pathogenesis of this disorder. He should have been aware about the hypothesis of Brissaud, as it was heavily circulated in the circles of neuropathologists in the France neuropathology at the start of the 20th century (Brissaud, 1895; Fleury, 1904). It is no doubt that the article from 1893 was known to him, and brought an additional argument for sustaining the conclusion so clearly stated by the Russian savant: “The results of our research lead us to say that there is an intimate relation between the substantia nigra and Parkinson’s disease. It most likely is a cause-effect link” (Lewy, 1923).

Tretiakoff drafted his thesis during the final years of the First World War when took place an outburst of encephalitis letargica. Constantin Alexander Economo Freiherr von San Serff, an Austrian neuropsychiatrist, showed that patients with this disorder had signs of Parkinson’s (von Economo, 1918). The anatomical pathology the brain in patients with encephalitis letargica was compared by Tretiakoff with the anatomical pathology of the brain in Parkinson’s disease, allowing him to draw his important conclusions. His research involved the examination of the substantia nigra in 54 cases, of which nine had paralysis agitans and three had postencephalitis. In seven cases (of which six with paralysis agitans) he identified a severe loss of pigmented nigral neurons, with edema of the cellular bodies, gummous degeneration, and neurofibrillary alterations. In surviving nigral cells, he identified Lewy bodies. In people with postencephalitis, he showed the presence of severe degeneration of the substantia nigra associated with hyaline and granular degeneration of the surviving cells (Tretiakoff, 1919). He concluded that changes in substantia nigra were recurrent in patients with Parkinson’s, and were associated with vascular pathology and various senile changes in other parts of the brain (Fleury, 1904). The thesis of Trietiakoff was initially regarded with disbelief. Only after a few years came the confirmation of his results through the works of other researchers like Foix and Nicolesco. In their monograph about the basal ganglia, published in 1925, they confirmed that substantia nigra in patients with Parkinson’s disease is severely altered, displaying neuronal atrophy, neurofibrillary changes, vacuolation, and Lewy bodies (Foix and Nicolesco, 1925).

Blocq and Marinesco presented their work during J.M Charcot’s lifetime. The presentation of the case was done at the Société de Biologie on 27 May 1893 when Charcot was still actively involved in running his program at Salpêtrière. Because Charcot held a very strict control over all material emanating from his service, it may be hard to credit major contributions to his students as independent investigators. During Charcot’s leadership, Salpêtrière became one of the most advanced centers of neuro-research in the world, through three main activities: development of scientific and teaching facilities, the characteristics of the staff and change in the composition of the patient pool (Micale, 1985). The facilities, teachers, and patients from Salpêtrière attracted numerous young, aspiring physicians and neuro-researchers from all over the world in the last quarter of the 19th century, leading to a high number of scientific breakthroughs (Micale, 1985). Charcot led all his pupils with a firm hand—each of his lectures was recorded and published in one of the several medical journals he founded (Ellenberger, 1970). The students worked in close collaboration with the master—the so-called charcoterie (Micale, 1985). Even the case published by Blocq and Marinesco was given to them by Charcot, as they acknowledge in the introduction of the manuscript (Blocq and Marinesco, 1893).

## Conclusion

This short discussion regarding the etiology of Parkinson’s disease shows how a single case report may play essential roles in the development of knowledge in biomedical sciences. Moreover, often the researchers who make a significant discovery, and are brought into the spotlight, are influenced by other researchers, with sometimes equally important roles. In the discovery of the association between lesions of substantia nigra and Parkinson’s disease, the article of Paul Blocq and Georges Marinesco had an enormous indirect contribution. Both actions, the publishing of the case report that stayed at the base for Brissaud’s theory, and the aid given by Marinesco to Tretiakoff in choosing his Ph.D. theme, are indirect, behind the scenes. However, without them, the connection would most likely be discovered much later, and subsequently, all advances in this field might have been delayed. Therefore, when researching the history of a scientific breakthrough, we must also look behind the researcher who won the spotlight and recognize the importance of the “back singers” too.

## Author Contributions

SH: performed historical analysis, analyzed the pathology information, drafted the initial form of the manuscript and approved the final version. ED: performed database searches, historical analysis, reviewed and rewrote parts of the manuscript, and approved the final version. OB: performed historical analysis, wrote parts of the manuscript and approved the final version.

## Conflict of Interest Statement

The authors declare that the research was conducted in the absence of any commercial or financial relationships that could be construed as a potential conflict of interest.
